# Spectrofluorimetric quantification of terconazole in pharmaceutical creams with comprehensive sustainability evaluation

**DOI:** 10.1186/s13065-026-01844-8

**Published:** 2026-06-06

**Authors:** Huda Salem AlSalem, Sara Naif Alharbi, Shimaa A. Mahmoud, Noha S. Katamesh, Mohamed A. Abdel-Lateef

**Affiliations:** 1 Department of Chemistry, College of Science, Princess Nourah bint Abdulrahman University, P.O. Box 84428, Riyadh, 11671 Saudi Arabia; 2 Pharmaceutical Analytical Chemistry Department, Faculty of Pharmacy (Girls), Al-Azhar University, Cairo, Egypt; 3 Department of Pharmaceutical Analytical Chemistry, Faculty of Pharmacy, Al-Azhar University, Assiut Branch, Assiut, 71524 Egypt

**Keywords:** Terconazole, Spectrofluorimetric determination, Eosin Y, Pharmaceutical cream samples, Greenness assessment

## Abstract

Terconazole is an effective antifungal drug with a broad spectrum of activity against many types of fungi. In the study, a spectrofluorimetric method was developed for the quantification of terconazole. Eosin Y, an efficient fluorescence probe, was used in the developed work. The developed method is based on forming a non-fluorescent association complex between terconazole and eosin Y, which quenches eosin Y’s inherent fluorescence intensity at both excitation and emission spectra. This quenching effect is highly correlated with the concentration of terconazole, with a linear range of 0.15–1.2 µg/mL. Furthermore, Stern-Volmer analysis was carried out to study the quenching fluorescence strength of eosin Y by terconazole. Moreover, the developed method exhibits an LOD of 0.041 µg/mL and a LOQ of 0.125 µg/mL. Moreover, the recommended method was verified in accordance with ICH guidelines. In addition, with respectable recovery values, the established procedures were successfully applied to the analysis of terconazole in vaginal cream dosage forms. Furthermore, the greenness of the entire proposed project was assessed using the following assessment tools: MoGAPI, Complex MoGAPI, MoGSA, BAGI, and RAPI. The green profiles provided outstanding evidence of the greenness and environmental friendliness of the proposed approach.

## Introduction

The global prevalence of fungal skin diseases caused by species such as Trichophyton and Candida has increased in recent years. These infections are more common among immunocompromised patients, including those receiving cancer chemotherapy, living with HIV infection, or undergoing organ transplantation [1]. Vulvovaginal candidiasis, mainly caused by Candida albicans, is one of the most common fungal infections and affects up to 75% of women of childbearing age at least once during their lifetime [2]. Fungal infections are commonly managed by oral and/or topical antifungal therapy [3]. Although oral systemic treatment is often effective, it may be associated with adverse effects and an increased risk of drug interactions. Terconazole (TCZ) is a recently developed antifungal drug with a wide range of effectiveness against many types of fungi. It falls under the category of triazoles, and it acts by inhibiting the function of the cytochrome P450 14-alpha-demethylase enzyme in Candida species, which results in the accumulation of sterol intermediates and reducing the concentration of ergosterol [4, 5]. Consequently, the synthesis pathways of ergosterol, a vital fungal sterol found in cell membranes, are disrupted [4, 5]. TCZ is pharmaceutically formulated as a topical therapy for treating vulvovaginal candida infection. Consequently, it is considered a widely used therapeutic option due to its broad-spectrum antifungal action and low side effects [4, 6].

Literature survey revealed that TCZ has been determined using various analytical techniques, including electrokinetic chromatography [7], HPLC-based methods [6, 8, 9], HPTLC [10], and UPLC [11, 12], in addition to a spectrofluorimetric method [13], electrochemical methods [5, 14], and spectrophotometric methods [8, 15]. Simple spectrometric methods remain attractive in pharmaceutical analysis owing to their accessibility, low cost, and suitability for routine quality control applications [16, 17]. One of these analytical tools is a spectrofluorimetric instrument, which is characterized by its superior sensitivity and selectivity for measuring pharmaceutical compounds [18, 19]. Xanthene dyes have been widely utilized in fluorescence probe-based spectroscopic techniques. Recently, they have been applied to determine several pharmaceutical compounds, including amiodarone, daclatasvir, clemastine, naftidrofuryl, cyclobenzaprine, and -blockers [20–24]. One of the common xanthene category dyes is eosin Y (ES-Y) dye, which has remarkable fluorescence properties in addition to its ability to form ion-pair complexes with the tertiary amine moiety of some active pharmaceutical ingredients. Due to TCZ having a tertiary amine moiety in its chemical structure, ES-Y is considered a desirable choice of fluorescence probes for the quantitation of TCZ. This work investigates the interaction between TCZ and ES-Y and its analytical application for determining TCZ. In addition, the alteration in the fluorescence intensity upon this reaction was exposed to Stern-Volmer analysis. Moreover, the reaction conditions of analysis will undergo optimization, and the validity of the developed method will be subject to validation according to ICH guidelines.

Green Analytical Chemistry (GAC) is based on environmental sustainability, which needs to be evaluated using a variety of complementary criteria that evaluate various ecological effect attributes. Five different greenness assessment tools were used to offer meticulous environmental characterization. Furthermore, the proposed procedure’s cost, usefulness, and sustainability were demonstrated by an extensive evaluation of its environmental impact using a variety of integrated innovative technologies, such as Modified Green Analytical Procedure Index (MoGAPI), ComplexMoGAPI, Modified Green Star Area (MoGSA), blueness, and redness tools. Although several analytical methods have been reported for the determination of TCZ, many of them rely on sophisticated instrumentation, involve relatively complex procedures, or may not be convenient for routine quality control laboratories. Therefore, there remains a practical need for a simple, rapid, sensitive, and cost-effective alternative method for the analysis of TCZ in pharmaceutical formulations. In the present study, a spectrofluorimetric method based on the quenching effect of eosin Y was developed for TCZ determination in vaginal cream samples. The proposed method was optimized, validated according to ICH guidelines, and further assessed using multiple sustainability metrics to demonstrate its practical and environmental suitability.

## Experimental

### Instrumentation

A Scinco fluorescence spectrometer (Scinco Co., Korea) fitted with a 150 W xenon arc lamp was employed to carry out the spectrofluorimetric measurements. A Jenway 350 pH meter was used to monitor the pH of the medium.

### Chemicals, reagents, and pharmaceutical samples

ES-Y (El-Nasr Co., Egypt) was dissolved and prepared in double-distilled water at a concentration of 30 mg/100 mL. The Teorell & Stenhagen (T&S) buffer was prepared [25–27]., and its pH was adjusted to the desired value using a calibrated pH meter. TCZ was kindly provided by Apex Pharm-S.A.E., Badr City, Egypt. 2-Propanol (chromatographic analytical grade) was produced by MERCK Co., Darmstadt, Germany. Gynoconazole 0.8%^®^ cream has been manufactured by Apex Pharm-S.A.E., Badr City, Egypt, and purchased from a local Egyptian pharmacy with batch number AR240286.

### Standard solution

Standard stock solutions of TCZ were prepared at a concentration level of 200 µg/mL by dissolving an appropriate amount (20 mg) in 100 mL of 2-propanol. Propanol was selected as a co-solvent owing to the limited aqueous solubility of TCZ and its ability to provide clear homogeneous solutions in the proposed reaction medium [28, 29]. In addition, TCZ working solutions were prepared by diluting the stock solution with water to obtain the desired concentrations for method analysis.

### Steps for fluorescence assay

Appropriate aliquots of TCZ working solution were transferred to a series of 10.0-mL volumetric flasks to obtain final concentrations of 0.15–1.2 µg/mL, followed by addition of 600 µL of T&S buffer (pH 3.5) and 800 µL of ES-Y solution. The flasks were mixed well, allowed to stand for 5 min, then completed to volume with distilled water. A reagent blank was simultaneously made under identical conditions for each experiment, except that TCZ was not added.

### Calibration graph construction

Fluorescence measurements were carried out at an excitation wavelength of 520 nm and an emission wavelength of 543 nm. The fluorescence intensity of Eosin Y in the absence (FI_0_) and presence ($\:{FI}_{TCZ}$) of terconazole was recorded. The calibration graph was constructed by plotting ΔFI against TCZ concentration (µg/mL), where $\:\varDelta\:FI=({FI}_{0}-\:{FI}_{TCZ})/{FI}_{0}$.

### Determination of TCZ in pharmaceutical cream

A suitable quantity (one gram) of Gynoconazole 0.8%^®^ cream was weighed and transferred into a clean volumetric glass container. An appropriate volume of 2-propanol was added, and the glass container was sonicated in an ultrasonic bath at a temperature of 50 °C for 20 min. The container content was completed to the mark with 2-propanol, allowed to stand to separate insoluble excipients, and the supernatant was subsequently filtered using a 0.45 μm membrane filter. The obtained solution was exposed to further dilution with water to obtain the required working solution, and the general analytical procedures were applied.

## Results and discussion

### Reaction mechanism and fluorescence spectra

**Scheme 1:**
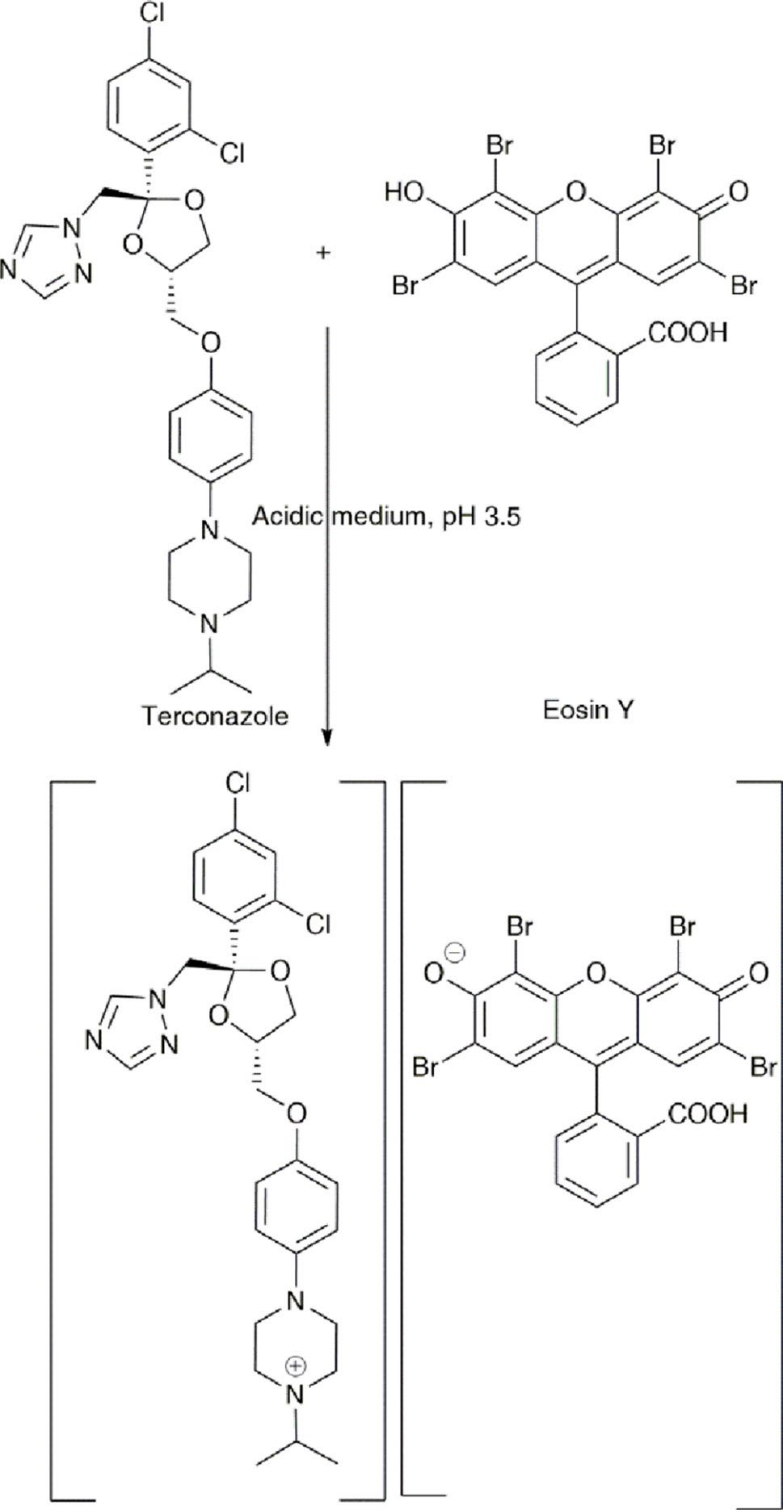
Proposed ion-pair association between protonated terconazole and anionic eosin Y under acidic conditions

**Fig. 1:**
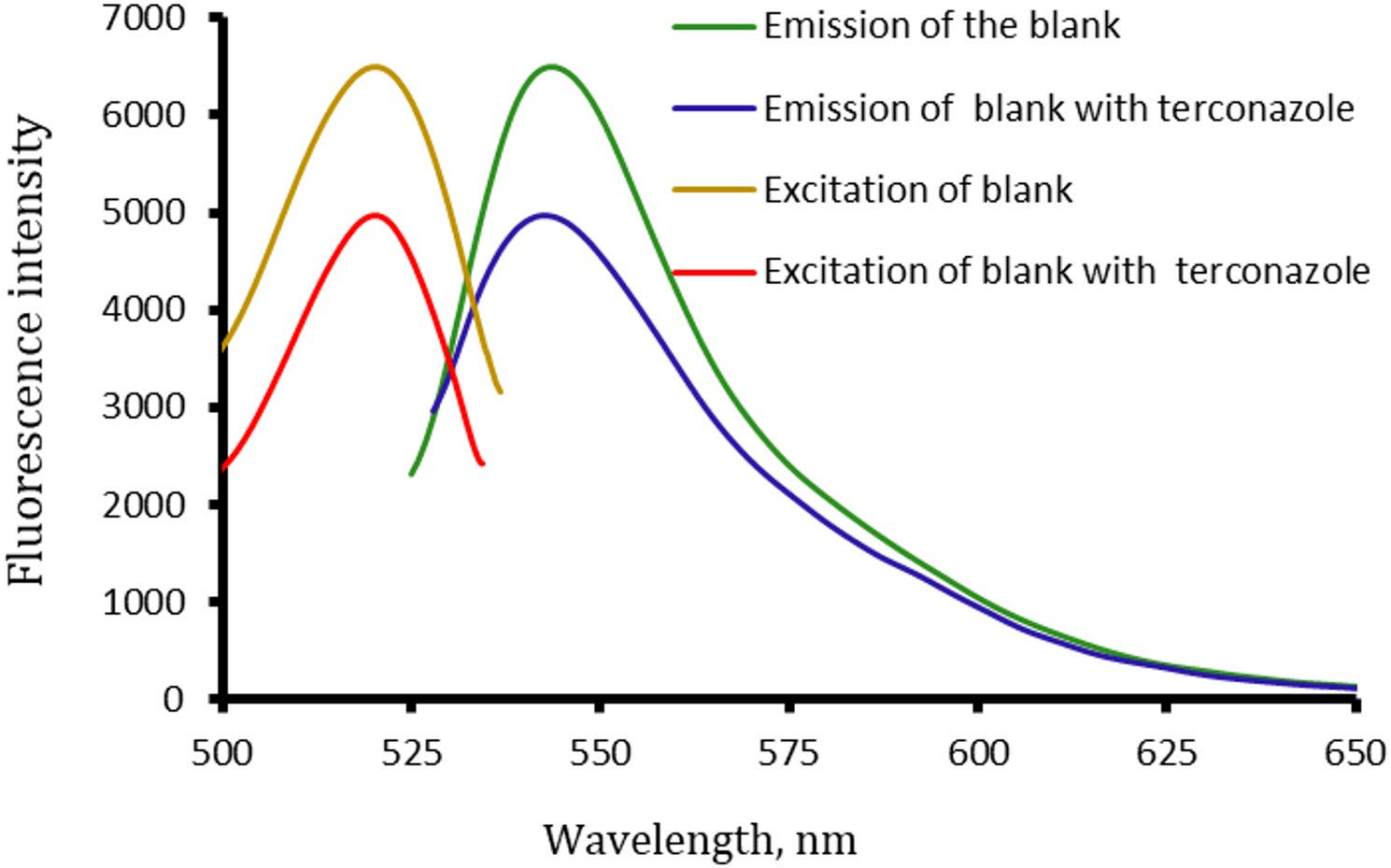
Fluorescence spectra of Eosin Y in the presence and absence of terconazole (1.0 µg/mL)

Due to its remarkable fluorescence properties (high quantum yield and photo-stability), ES-Y is a desirable choice as a spectroscopic probe widely utilized in quantitatively determining many nitrogenous compounds (especially tertiary amines) in an acidic medium. Because TCZ contains tertiary amine functionality, it can be protonated under acidic conditions. Thus, ES-Y can form ion-pair complexes with cationic species, and it is a viable candidate for interacting with the positively charged TCZ molecule, as shown in Scheme 1 [21, 22, 30–33]. In addition to the presence of a basic nitrogenous center in the analyte as an essential requirement for the formation of an ion pair complex with ES-Y, the reported study correlated the formation of the ES-Y-analyte association complex with requiring a sufficient degree of lipophilicity in the analyte [21], however in the current study, TCZ is classified as a lipophilic compound and has a log P value of 5.37 [34] which indicates its ability to form a binary complex with ES-Y reagent. Therefore, hydrophobic forces and electrostatic attraction cause the complex to develop between the protonated TCZ and the eosin anion. As shown in Fig. 1, the quenching effect of TCZ on the fluorescence intensity of the ES-Y reagent was observed in both the excitation (520 nm) and emission (543 nm) of the reagent. The degree of quenching effect is correlated with the concentration level of TCZ in a specified range.

### Optimization of the reaction variables

#### pH of the reaction

**Fig. 2:**
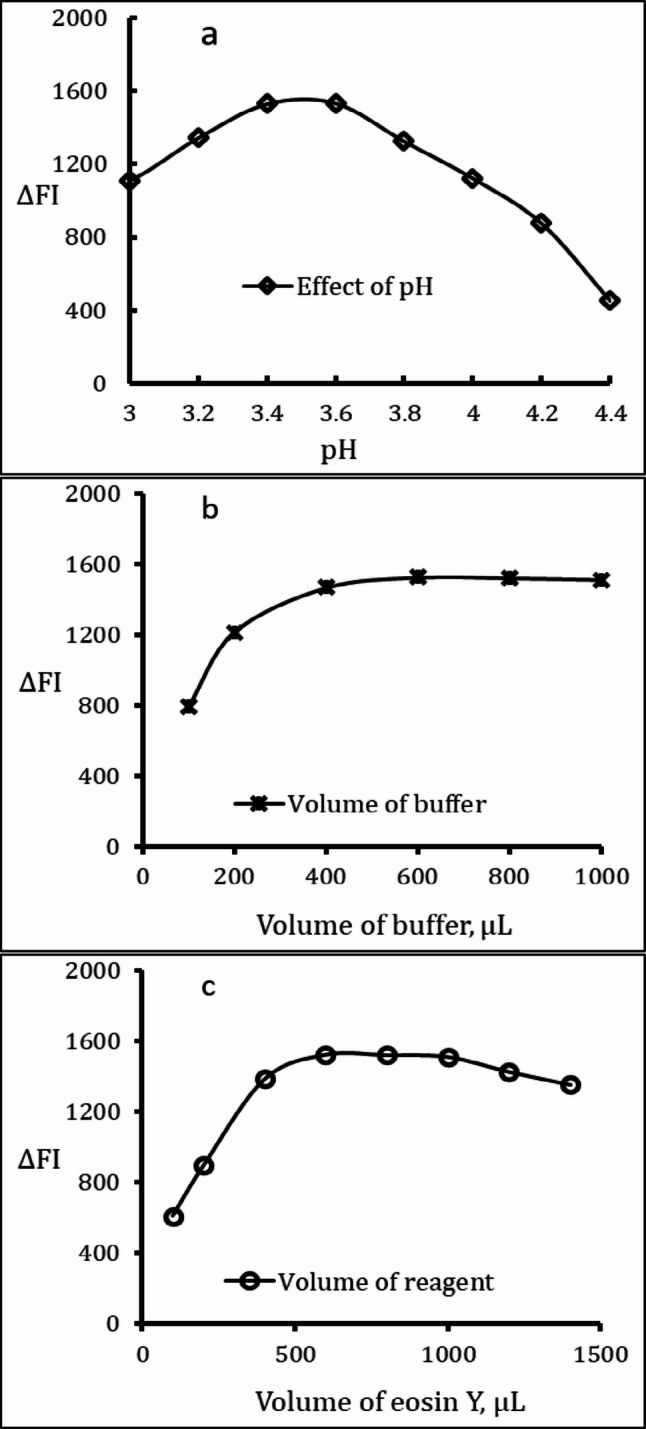
Optimization of variables affecting the quenching effect of TCZ (1.0 µg/mL) on the fluorescence intensity of the blank, including pH (**a**), volume of T&S buffer (**b**), and volume of Eosin Y solution (0.3 mg/mL) (**c**)

Different pH values were tested to reach the highest quenching effect by TCZ on the FI_0_ of ES-Y. The highest quenching effect by TCZ was achieved at the pH range of 3.4–3.6, Fig. 2a. Different buffer solutions were also tested at the pH value of 3.5 using T&S, citric acid buffer, and acetate buffer solutions. The results demonstrated that T&S was superior and had better results than other buffer solutions, and that T&S was the ideal pH range for determining TCZ. In addition, the impact of the buffer volume on complex formation was examined throughout a range of 100–1000 µL of T&S buffer solution. The greatest quenching FI_0_ value was recorded with quantities of 600 µL of T&S buffer, Fig. 2b.

#### Volume of ES-Y and diluting solvent

As seen in Fig. 2c, the study investigates the impact of ES-Y volume on the quenching FI_0_ intensity of the final solution. The results showed that as the concentration of ES-Y increased, the quenching FI_0_ value progressively increased as well. The quenching in FI_0_ attained its optimum values at 600 µL of the used ES-Y and stayed with minor variations between 600 and 1000 µL. The reaction would be fully incomplete if there was insufficient quantity of ES-Y, whereas ΔFI would be reduced if there was too much ES-Y. Thus, 800 µL was determined to be an appropriate eosin volume in the current study. The reaction product was diluted using water, absolute ethyl alcohol, methylene chloride, and acetone solvents; their effects on complex formation and, accordingly, on the resulting ΔFI values were examined. Distilled water was the most suitable solvent for dilution since it yielded the highest quenching values, while the other solvents produced lesser values.

#### Addition order, stability, and reaction time

After some trials for the current experiment, the addition sequence selected was TCZ solution first, T&S buffer second, and ES-Y solution, consecutively. The mixing of solutions at room temperature was followed immediately by creating the complex. After examining the effect of standing time, the interaction between TCZ and ES-Y was found to occur immediately upon mixing the reactants at room temperature. No significant changes in ΔFI values were observed with increasing standing time, and the measured response remained stable for at least 2 h. Therefore, a fixed waiting time of 5 min was adopted only to ensure complete mixing and uniform measurement conditions throughout the analysis.

### Validation of the recommended method and Stern-Volmer analysis

The International Council for Harmonization’s (ICH) guidelines were followed in the analytical validation of the recommended method [35].

#### Linear range and statistical parameters

Following the process of creating calibration curves by graphing ΔFI values against the concentration of TCZ (µg/mL), the linear range of the recommended method was 0.15–1.2 µg/mL, combined with a regression equation of y = 0.2291x + 0.0071 and r^2^ = 0.9989. Moreover, Table 1 provides the other computed statistical data for the built calibration curve.

**Table 1:** Analytical parameters of the proposed method

Parameter	Result
Linear range µg/mL	0.15–1.2
Slope	0.229
SD of slope (S_b_)	3.7 × 10^⁻³^
Intercept	0.007
SD of intercept (S_a_)	2.87 × 10^⁻³^
r	0.9994
r^2^	0.9989
LOQ	0.125 µg/mL
LOD	0.041 µg/mL

#### The quenching constant Stern-Volmer analysis

The quenching of the inherent fluorescence intensity of ES-Y by TCZ has been studied by using Stern-Volmer analysis as:

$$\:\frac{{FI}_{0}}{{FI}_{TCZ}}={K}_{SV}\left[Q\right]+1$$

where $\:{K}_{SV}$ refers to Stern-Volmer quenching constant, and [Q] refers to the concentration of TCZ in molar. It was found that the fluorescence intensity ratio was stated as a satisfactory linear correlation (*r* = 0.995), the $\:{K}_{SV}$ value is equal to 176,881 M^−1^, and an intercept value of 0.98, which is consistent with the above-mentioned linear range for determining TCZ by ES-Y using the recommended method.

#### LOD and LOQ

The limits of detection (LOD) and quantification (LOQ) were calculated according to ICH guidelines using the equations: LOD = 3.3σ/S and LOQ = 10σ/S, where σ is the standard deviation of the intercept of the regression line and S is the slope of the calibration curve. The calculated LOD and LOQ values were 0.041 and 0.125 µg/mL, respectively.

#### Accuracy and precision

The accuracy of the proposed method was evaluated at three concentration levels of TCZ standard solution (0.25, 0.5, and 1.0 µg/mL). The obtained recovery values ranged from 98.52 to 101.34%, indicating satisfactory accuracy of the method (Table 2). Values are expressed as mean of three replicate measurements.

**Table 2:** Accuracy data of the proposed method

Concentration of TCZ (µg/mL)	Recovery (%) ± SD*
0.25	99.7 ± 1.73
0.5	101.34 ± 1.41
1.0	98.52 ± 1.25

*Values are mean of three replicates

The precision of the proposed method was independently assessed using separate replicate analyses at the same concentration levels under intra-day and inter-day conditions. The %RSD values ranged from 1.37 to 1.98%, confirming good repeatability and intermediate precision of the proposed procedure (Table 3). Values are based on three independent replicate measurements for each concentration level; %RSD was calculated from these replicates.

**Table 3:** Intra-day and inter-day precision data for the proposed method

Assay	Concentration of TCZ (µg/mL)	Mean recovery (%)	%RSD
Inter-day	0.3	98.46	1.86
	0.6	101.20	1.37
	0.9	100.99	1.65
Intra-day	0.3	101.59	1.98
	0.6	99.82	1.91
	0.9	101.34	1.73

Values are mean of three replicates; %RSD was calculated from these replicates

#### Robustness

The robustness of the recommended method was evaluated by examining the impact of minor adjustments to the experimental parameters (buffer volume, pH, and ES-Y volume) on the calculated ΔFI values. Each variable was given this straightforward adjustment independently, with the other variables being kept constant. The resilience and dependability of the recommended spectroscopic procedures were found to be demonstrated by the observation that even a small alteration to these experimental conditions did not result in a flaw in the ΔFI values, Table 4.

**Table 4:** Evaluation of robustness of the proposed method

Variable	% Recovery ± SD*
Volume of reagent	
750 µL	98.7 ± 1.19
850 µL	101.52 ± 1.06
Volume of T&S buffer	
550 µL	99.44 ± 0.75
650 µL	100 ± 1.9
pH value	
3.4	98.64 ± 1.16
3.6	101.46 ± 0.88

* TCZ concentration is 1.0 µg/mL

### Application on pharmaceutical vaginal cream samples

The content of TCZ in Gynoconazole 0.8%^®^ vaginal cream was analyzed using the recommended method. After applying the recommended method to determine TCZ, the mentioned sample yielded a mean recovery percentage ± SD of 102.34 ± 2.02. It was concluded that there were no notable distinctions between the reported method and the recommended method concerning accuracy and precision.

### Comparison with relevant reported methods

A comparison of the proposed method with relevant reported analytical procedures for terconazole determination demonstrates its practical merits. The previously reported UV spectrophotometric method showed linearity over the concentration range of 50–250 µg/mL for bulk terconazole, indicating a substantially higher working concentration range than the present procedure [15]. A micellar liquid chromatographic method applied to bulk drug, dosage forms, and spiked plasma exhibited linearity over 16–80 µg/mL for terconazole with a reported limit of detection of 1.67 µg/mL, but it required chromatographic instrumentation and a multicomponent mobile phase [9]. In another study, RP-HPLC, derivative spectrophotometric, and chemometric methods were proposed for the simultaneous determination of terconazole with benzoic acid in cream formulations, with reported linear ranges of 4–128, 5–40, and 3–11 µg/mL, respectively [8]. In contrast, the present eosin Y-based spectrofluorimetric method provides a lower working concentration range (0.15–1.2 µg/mL) with a detection limit of 0.041 µg/mL, while offering operational simplicity, rapid analysis, low solvent consumption, and direct applicability to pharmaceutical cream samples. Therefore, the proposed method represents a sensitive and practical alternative for routine quality control analysis of terconazole formulations, in addition to its favorable sustainability characteristics.

### Evaluation of the sustainability of the proposed method

In laboratory settings, the principles of “green chemistry” have attracted considerable attention. Use of sophisticated evaluation tools is necessary for precisely assessing the environmental impact of chemical operations [36, 37]. Understanding how environmentally friendly chemical processes are performed is just as essential as creating the regulations. Metrics offer a measurable way to evaluate how chemical activities affect the environment and point out areas that need adjustment. Chemical labs can implement safer, more environmentally friendly, and sustainable practices through implementing these parameters [38]. In order to ensure safer manufacturing and use, the developed metrics assess the toxicity and hazards associated with chemicals.

#### Greenness assessment

##### MoGAPI tool

**Fig. 3:**
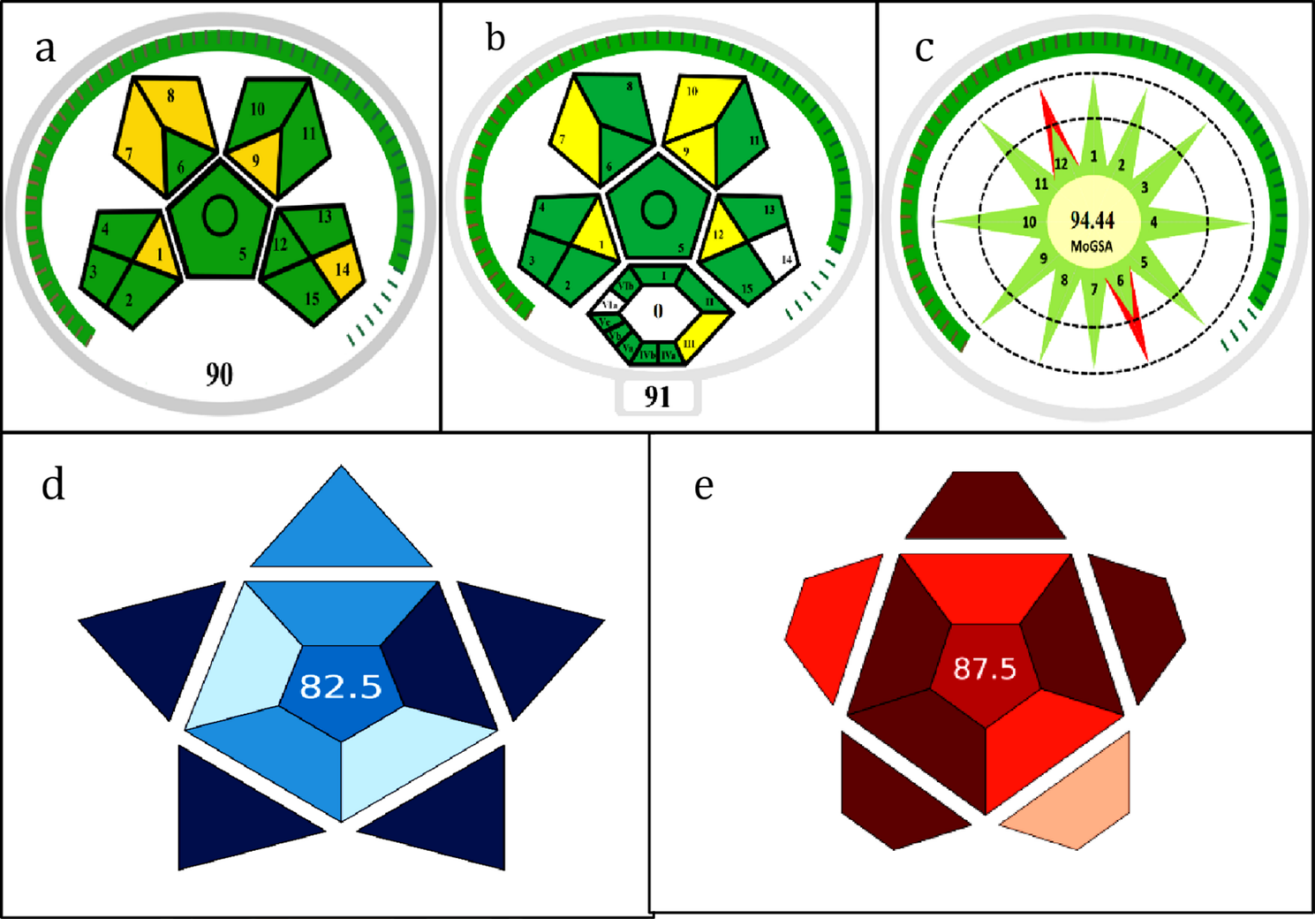
Different tools for assessment of the sustainability of the current work, including MoGAPI (**a**), Complex Mo-GAPI tool (**b**), MoGSA tool (**c**), BAGI tool (**d**), and RAPI tool (**e**)

More insightful and instructive metrics, including the newly introduced Modified Green Analytical Procedure Index (MoGAPI) tool in September 2024 [39], have replaced the common GAPI metric. This recently released metric is similar to the traditional GAPI in that it evaluates the entire analytical approach by evaluating 15 distinct particular elements a single at a time, beginning with sampling and continuing through the method category (quantitative or qualitative), preparation of the samples, volume of reagents and solvents, their risks and energy consumption, until it reaches the volume of waste generated, and any potential waste treatment. Additionally, it improves the conventional GAPI in terms of being quickly and easily computed with open-source software. Following the application of this statistic to the developed approach, the approach has achieved the highest green method score of 90, as represented in Fig. 3a.

##### Complex MoGAPI tool

To improve the assessment procedure even more, ComplexGAPI, a supplementary tool, was released in 2021 [40]. An extra hexagonal segment has been added to the original GAPI diagram in ComplexGAPI to symbolize the steps taken prior to sample preparation and the final analysis. This additional hexagon facilitates the evaluation of sustainability in a number of areas, such as production yields and conditions, solvent and reagent selection, equipment and technique use, and purifying procedures. A modified tool called ComplexMoGAPI was introduced to satisfy the needs of ComplexGAPI users. It combines the accurate overall results of ComplexGAPI with its appealing design. ComplexMoGAPI offers an exhaustive evaluation of sustainable development methods and enables a more precise and independent evaluation of methods. The associated software streamlines the application process, resulting in quicker and easier investigations. The application can be found on bit.ly/ComplexMoGAPI as an open-source project. According to Fig. 3b, this technique received a perfect environmentally friendly score of 91.

##### MoGSA tool

An innovative metric, MoGSA, and software were utilized to evaluate how environmentally friendly chemical processes were in laboratory environments. MoGSA improves on the conventional Green Star Area Index (GSAI) by enabling users to apply particular Green Chemistry principles only when they are essential to the chemical process under consideration. The shortcomings of GSAI are addressed by this method, which frequently fails to distinguish between green and non-green practices and fails to take into consideration how the 12 Green Chemistry principles vary in their relevance in various situations [41]. The software is currently available at https://bit.ly/MOGSA. This approach yielded a MoGSA score of 94.4, which, as shown in Fig. 3c, indicates a high degree of ecological friendliness.

#### Blueness assessment

The Blue Applicability Grade Index (BAGI) was employed to evaluate the practical sustainability of the proposed method [42, 43]. BAGI is a comprehensive metric that considers ten key operational parameters, including the type of analysis, number of chemical components, instrumentation, sample handling efficiency, sample collection, throughput (samples per hour), reagents and materials used, degree of automation, preconcentration requirements, and total number of samples processed. These criteria collectively provide an integrated assessment of the method’s practicality and sustainability. The developed method achieved a BAGI score of 82.5 (Fig. 3d), indicating a high level of operational efficiency and practical applicability, particularly in terms of throughput, automation potential, and cost-effectiveness.

#### Redness assessment

The Red Analytical Performance Index (RAPI) [44]was applied to evaluate the analytical performance of the proposed method. RAPI is a recently introduced assessment tool designed to comprehensively examine analytical methods with particular emphasis on statistical and validation-related characteristics. The selection of evaluation criteria was guided by the International Council for Harmonization (ICH) validation guidelines, widely accepted analytical principles, and good laboratory practices. Since analytical performance is influenced by multiple factors, fundamental and universally applicable validation parameters were considered to ensure an objective assessment. Similar to the BAGI approach, RAPI employs a star-shaped graphical representation; however, its scoring system differs in terms of performance visualization. Each criterion is represented using a graded color intensity scale ranging from white (score 0) to dark red (score 10). The color mapping scheme is based on the “Reds” colormap implemented in the Matplotlib module [45], enabling clear visual interpretation of analytical performance. The developed method achieved an RAPI score of 87.5 (Fig. 3e), reflecting strong analytical performance in terms of linearity, validation parameters, robustness, and overall methodological reliability.

## Conclusion

In the study, the spectrofluorimetric technique has been used to quantify TCZ. The studied drug can form an ion-association complex with ES-Y dye in an acidic state through an electrostatic attraction and hydrophobic force interaction. As a result, the ES-Y dye’s fluorescence spectra were markedly quenched, and a new spectrofluorimetric method was developed to quantify TCZ. The developed method is distinguished by its sensitivity, ease of use, low cost, and quickness of methodology. Furthermore, the developed method did not include any complicated sample preparation procedures, a long analysis time, or extraction compared with the chromatographic methods, and it has good sensitivity to the spectrophotometric methods in the determination of TCZ. As a result, the recommended methods were used to analyze TCZ in pharmaceutical samples, which have potential applications in quality control. Owing to the green evaluation that was carried out utilizing the MoGAPI, Complex MoGAPI, MoGSA, BAGI, and RAPI tools in an attempt to implement an environmentally friendly approach, the obtained greenness profiles demonstrated the environmental suitability of the proposed method. This illustrates the approach’s commitment to environmentally sustainable practices, which makes it a suitable option for pharmaceutical analysis.

## Data Availability

All data generated or analyzed during this study are included in this published article. Additional data are available from the corresponding author on reasonable request.

## Abbreviations

TCZ: Terconazole
ES-Y: Eosin Y
FI: Fluorescence intensity
ΔFI: Difference in fluorescence intensity
LOD: Limit of detection
LOQ: Limit of quantification
ICH: International Council for Harmonisation
T&S: Teorell and Stenhagen buffer
MoGAPI: Modified Green Analytical Procedure Index
MoGSA: Modified Green Star Area
BAGI: Blue Applicability Grade Index
RAPI: Red Analytical Performance Index
